# Supplementary material for: The causal relationship between the human gut microbiota and pyogenic arthritis: a Mendelian randomization study
Source: Front Cell Infect Microbiol. 2024 Nov 26;14:1452480. doi: 10.3389/fcimb.2024.1452480 (PMC11629706; doi:10.3389/fcimb.2024.1452480)
Supplement: Supplementary file 1 [file DataSheet1.zip › mendelian test/ebi-a-GCST90027757.csv_leave_one_out.pdf]

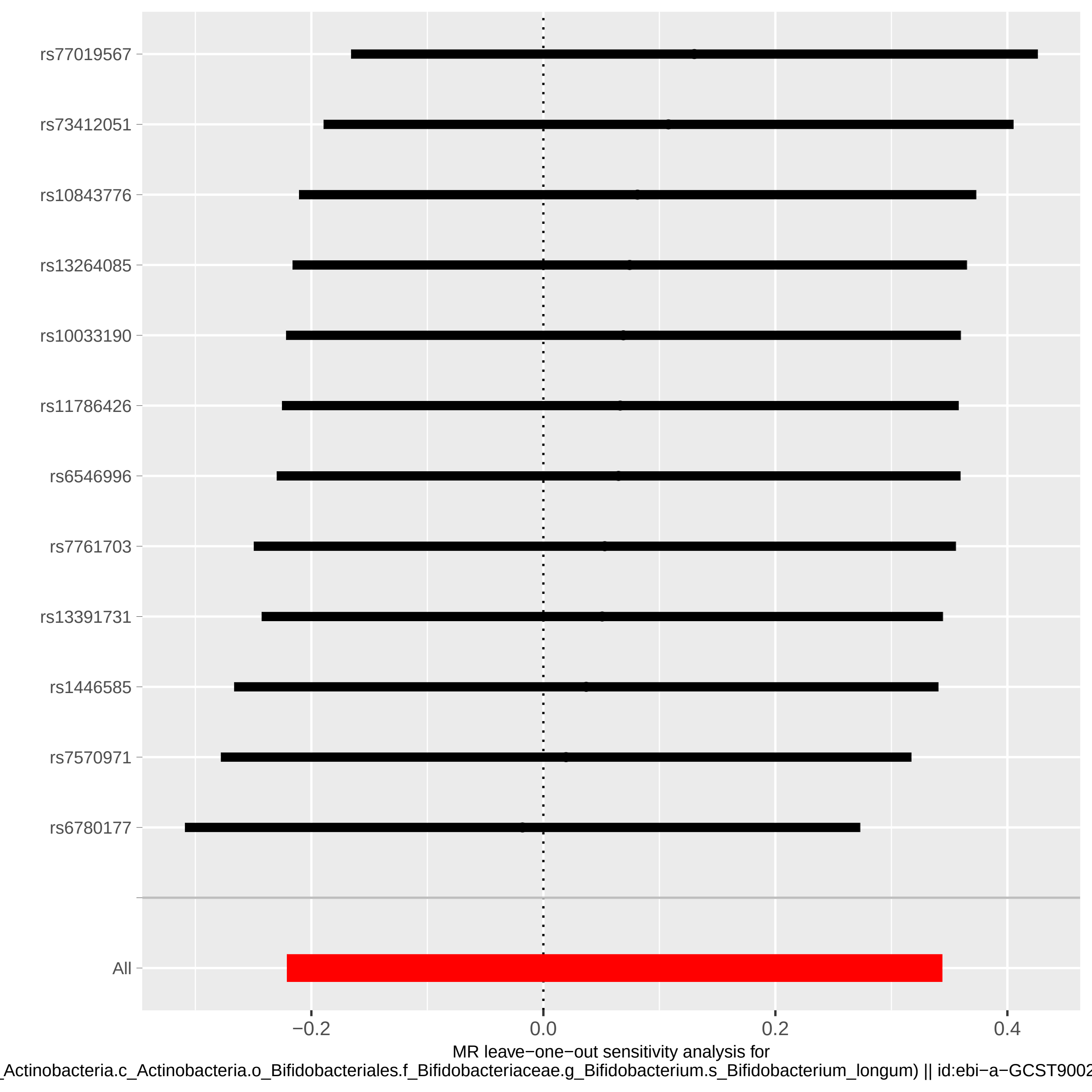

rs77019567
rs73412051
rs10843776
rs13264085
rs10033190
rs11786426
rs6546996
rs7761703
rs13391731
rs1446585
rs7570971
rs6780177
All
−0.2
0.0
0.2
0.4
MR leave−one−out sensitivity analysis for
_Actinobacteria.c_Actinobacteria.o_Bifidobacteriales.f_Bifidobacteriaceae.g_Bifidobacterium.s_Bifidobacterium_longum) || id:ebi−a−GCST9002
